# Supplementary figures and images for: Deep audio embeddings for vocalisation clustering
Source: PLoS One. 2023 Jul 10;18(7):e0283396. doi: 10.1371/journal.pone.0283396 (PMC10332598; doi:10.1371/journal.pone.0283396)

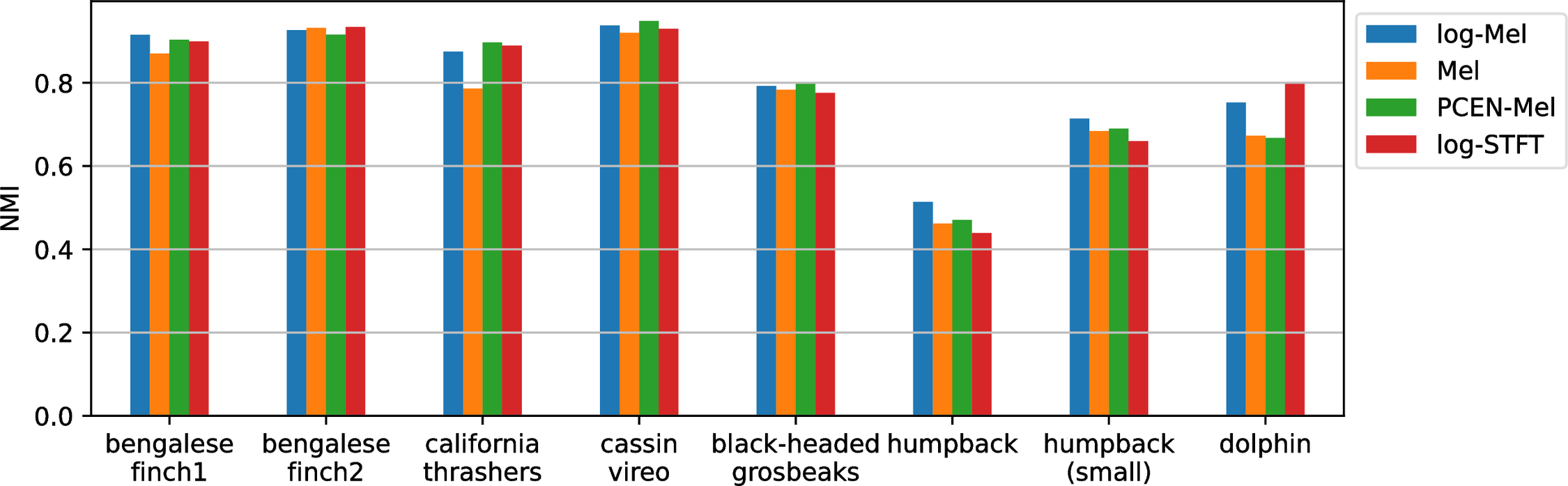

Supplement: S1 Fig — (TIF) [file pone.0283396.s001.tif]
